# Wild Plants Drive Biotic Differentiation Across Urban Gardens

**DOI:** 10.1002/ece3.71527

**Published:** 2025-06-04

**Authors:** Aaron N. Sexton, Felix Conitz, Ulrike Sturm, Monika Egerer

**Affiliations:** ^1^ Urban Productive Ecosystems, School of Life Sciences Technical University of Munich Freising Germany; ^2^ Museum für Naturkunde Berlin—Leibniz Institute for Evolution and Biodiversity Science Berlin Germany

**Keywords:** beta diversity, biotic homogenization, community composition, community ecology, community gardens, urban ecosystems, urbanization

## Abstract

The Urban Biotic Homogenization (UBH) hypothesis predicts that ecological communities should become increasingly similar in their species composition across an urbanization gradient, and that spatially independent cities should be more similar in species composition to each other than they are to their surrounding rural communities. However, there is conflicting scientific evidence for these predictions. Using 4 years of plant community data in 30+ urban community gardens across two European cities, we find no support for this hypothesis, especially among spontaneous, or wild plant species. Wild plant species exhibited increased biotic differentiation with urbanization (i.e., increased beta diversity), and plant species community composition was significantly different between the two cities. Wild plant species contributed more to overall beta diversity compared to cultivated plant species. These findings suggest that wild plant species may combat urban biotic homogenization in garden ecosystems and should therefore be supported to increase urban ecological heterogeneity.

## Graphical abstract

In this study, we directly test the Urban Biotic Homogenization (UBH) hypothesis using 4 years of data on plant communities in 30+ community gardens across two large European cities, Berlin & Munich. Here, we find no evidence to support either of the UBH hypotheses, especially among wild plant species. Beta diversity of wild plant species increased with urbanization, and across the wild and full plant communities, the two cities exhibit significantly different community composition.

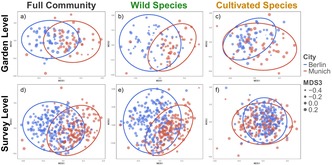

## 1 Introduction

The Urban Biotic Homogenization (UBH) hypothesis posits that with increasing urbanization comes increasing homogenization of ecological communities (McKinney 2006). In his original presentation of this hypothesis, McKinney suggested that urbanization is a central driver of biotic homogenization globally. Since then, this theory has dominated urban ecological theory. According to the UBH hypothesis, we would expect that beta diversity, the variation among local biodiversity, decreases along an urbanization gradient, generating increased biotic homogenization. Equally, the community composition of two spatially independent cities should be more similar to each other than they are to their surrounding rural communities. There have been several studies, especially among bird communities, that have found support for the UBH hypothesis (e.g., Deng et al. 2025; Clergeau et al. 2001). Studies have found lower levels of beta diversity in urban bird communities compared to communities in natural settings, and that the composition of urban communities is distinctly different from their surrounding rural communities (Colléony and Shwartz 2020; Sidemo‐Holm et al. 2022). Similar findings have been documented in other taxa including plants (Schwartz et al. 2006) and nocturnal and flying insects (Merckx and Van Dyck 2019).

While the UBH hypothesis has been influential for much of the discussion and research regarding urban ecology, there is conflicting evidence for this hypothesis. A 2022 synthesis (Lokatis and Jeschke 2022) found that across 225 individual studies, the UBH hypothesis was only supported 55% of the time. Several studies have even documented increases in biotic differentiation in urban ecosystems. For example, Aronson et al. (2014) found the beta diversity of woody species to increase with urbanization in the New York metropolitan region. A recent global synthesis found both taxonomic and phylogenetic beta diversity of bee communities to increase with urbanization (Tsang et al. 2025). Another study found increased inter‐population genetic differences in urban populations of the common ragweed ( *Ambrosia artemisiifolia* ) compared to rural populations, which the authors attributed to increased urban habitat heterogeneity (Gorton et al. 2018). A 2024 study in Brazil found the beta diversity of plant‐herbivore interactions to be high across an urban landscape and to have no negative relationship with urban intensity, calling the UBH hypothesis into question (de Araújo et al. 2024).

Altogether, it may be that the UBH hypothesis is only supported under certain scenarios, habitat types, or taxa. Urban ecosystems are highly heterogeneous, and there may be systems that are overlooked in their contribution to overall urban biodiversity (Band et al. 2005). For example, an extensive survey of all vegetation patches in Zurich, Switzerland found that 75% of patches were < 20 m^2^ in size, and that these small patches hosted greater beta diversity than large patches—which held consistent even for less common species (Vega and Küffer 2021). Overlooked habitats such as small vegetation patches, or taxa such as *Ambrosia artemisiifolia* mentioned above, exhibit increased differentiation in urban ecosystems. Without a full accounting of such habitats or species, support for the UBH phenomenon may be overestimated. Another ecosystem that has been overlooked in this regard is urban community gardens (Egerer et al. 2024). Urban community gardens have been shown to host high degrees of both cultivated and wild, or spontaneous (i.e., naturally occurring and not purposefully planted) biodiversity (Seitz et al. 2022). This balance of taxa makes urban community gardens an especially interesting habitat to study both cultivated and wild plant species—their coexistence and community composition—as compared to an urban park which tends to be dominated by cultivated and managed species, or ruderal habitats which are dominated by wild species.

However, there has not yet been an investigation into how urbanization influences their beta diversity and community composition across spatial and temporal scales and between cities. Finally, urban community gardens are a valuable testing field to address the UBH hypothesis across cities as they represent similar habitat types that can be directly compared within and across cities.

In this study we ask the broad question: Do we see support for the UBH hypothesis in urban community gardens, and if so, for which species? Specifically, we ask: (Q1) Do plant communities in urban community gardens become more homogeneous along an urbanization gradient, and (Q2) Are the plant communities from two distant cities highly similar, or are they unique from one another? To build on these questions and identify the drivers of their results, we also ask: (Q3) Is homogenization (both within and across cities) equal among wild and cultivated plant species? and (Q4) Do wild and cultivated plant species contribute equally to overall beta diversity? To answer these questions, we utilized 4 years of plant surveys in community gardens across two cities (Berlin & Munich, Germany) spanning from rural to highly urban in their landscape surroundings (Egerer et al. 2024).

We hypothesized a lack of support for the UBH hypotheses in community gardens, due to both the heterogeneity of urban landscapes (Niemelä 1999) and the influence of other drivers on plant communities such as historical urban development and gardener plantings in these social‐ecological systems (Egerer et al. 2024). Specifically, we predicted that (i) beta diversity would increase along an urbanization gradient (i.e., increased biotic differentiation with urbanization) across the entire plant community, and that (ii) this effect would be strongest for wild species. Among cultivated species, we predicted (iii) no relationship between urbanization and beta diversity, as they are determined by gardeners and their motivations and not necessarily environmental factors (Kendal et al. 2012; Egerer et al. 2019). Additionally, we predicted that (iv) both the wild and cultivated plant communities would be distinct between the two cities due to regional, climatic, and cultural differences between Berlin and Munich. Finally, we predicted that (v) wild species would have a wider species pool and therefore contribute more to overall beta diversity in the gardens.

## 2 Methods

### 2.1 Study System

**FIGURE 1:**
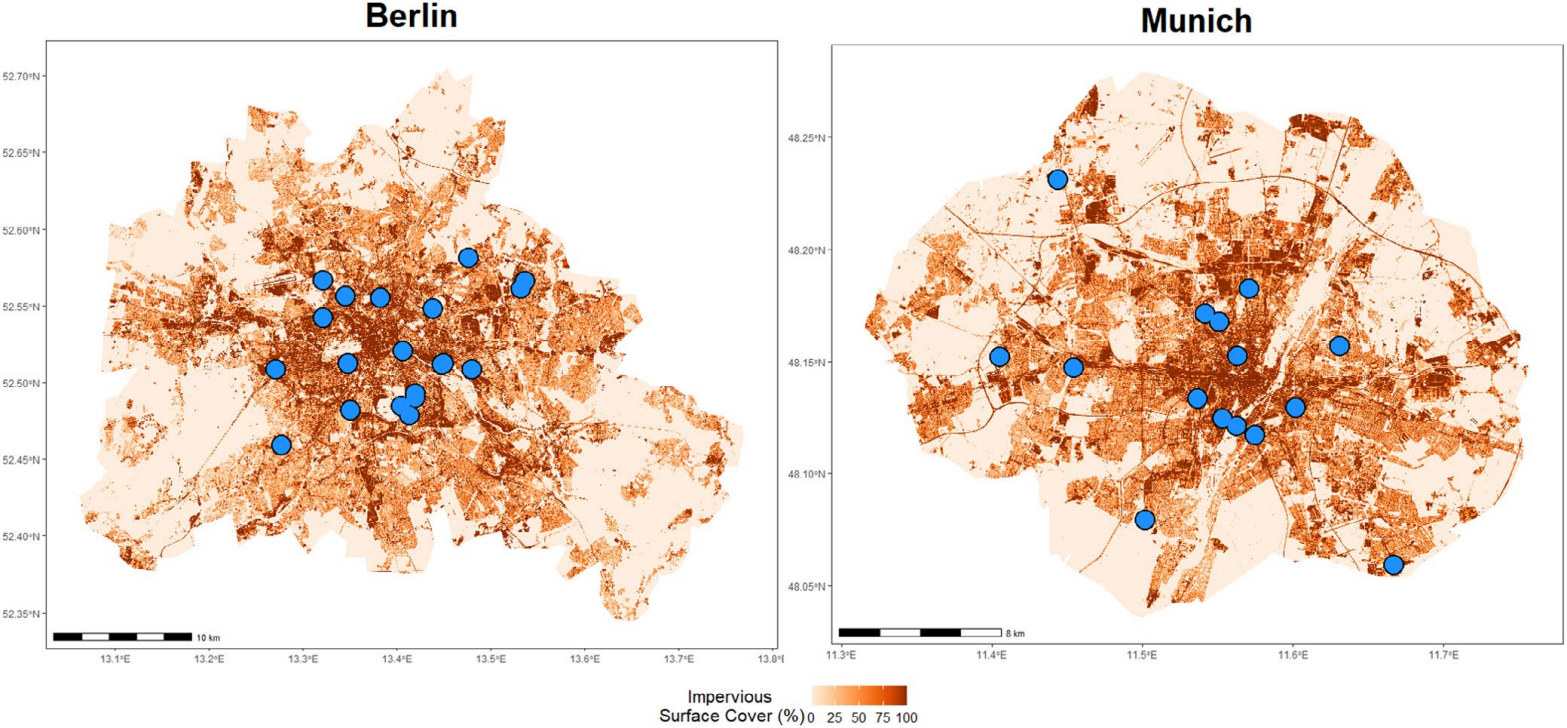
Map of all community garden study sites in blue points. Percentage impervious surface, here our metric of urbanization, is shown in orange to dark red.

We tested our hypotheses in 33 urban community gardens (Figure 1) in Berlin (52.5200° N, 13.4050° E) and Munich (48.1351° N, 11.5820° E), Germany over 4 years (2020–2023). The gardens were selected to represent variety in both biophysical characteristics as well as sociocultural characteristics of gardeners, to cover the range of habitat characteristics, landscape context, gardener motivations, and management practices that may influence plant species composition. The gardens vary in size (0.04–2.39 ha) and location along a landscape urbanization gradient (9.6%–96.2% landscape imperviousness within a 1000 m buffer). We calculated urbanization (i.e., landscape imperviousness) within a 1 km buffer surrounding each garden (von Der Lippe et al. 2020). We used the Copernicus High Resolution Layer: Imperviousness Density (IMD) 2018 (European Union, Copernicus Land Monitoring Service 2020, European Environment Agency (EEA)) with a resolution of 10 × 10 m. Landscape imperviousness was calculated in ArcGIS Pro (ESRI) using the zonal statistic tool. 1000 m was selected as it is a common buffer used in urban ecological studies, especially those focusing on plant‐pollinator interactions (e.g., Wilson and Jamieson 2019; Braman et al. 2023; Buchholz and Egerer 2020). Garden size, imperviousness, and the methods for imperviousness calculation are provided in Data S1. The inclusion of sites with low impervious cover allows us to cover the entire urbanization gradient and compare more rural versus more urban sites, which is a bias many UBH studies do not address (Lokatis and Jeschke 2022). Berlin & Munich experience climatic differences, particularly in annual precipitation, with Berlin generally drier (mean annual precipitation of 580.1 mm) than Munich (mean annual precipitation of 939.7 mm), and the two cities having relatively equal temperatures (10.7°C and 10.1°C mean annual temperatures respectively from 1991 to 2020; Deutscher Wetterdienst 2023).

### 2.2 Plant Surveys

We conducted plant surveys four times per year in each garden during the primary growing season in these gardens (April–September). Within each garden, we established a 400 m^2^ (20 × 20 m or 40 × 10 m to accommodate narrower gardens) study plot in the center of each garden in which to concentrate our data collection. For two gardens slightly less than 400 m^2^, we expanded the sampling area to reach the 400 m^2^ criteria, including buffering areas around the garden (e.g., sidewalks, hedges) to maintain the sampling size, thus adding a buffer area of 50–90 m^2^. We randomly placed eight 1 × 1 m sampling quadrats within the 400 m^2^ study plots in which we identified the species identity of all plant species (see: Neumann et al. 2024; Seitz et al. 2022). In the case of completely bare substrate or impervious surface, zero plant species were recorded. In addition, we recorded whether the species was a cultivated (i.e., crops and ornamentals) or wild species (spontaneous and naturally spreading species including native and weedy species). Cultivated versus wild determinations were made by our team of researchers and expert botanists, largely following the protocols determined by Seitz et al. (2022). The full species list and their cultivated/wild determinations are provided in Data S2.

### 2.3 Statistical Analyses

We calculated beta diversity at two scales: (i) the survey, and (ii) the garden (all four survey rounds of each year pooled). These two scales cover spatial differences (subplot variation vs. garden‐wide variation) and temporal differences (seasonal differences at the survey level vs. entire species pool at the garden level), henceforth termed “spatio‐temporal scales”. Beta diversity was calculated as a multivariate homogeneity of group dispersions (or variances), with surveys, or gardens as groups. Variances were calculated based on a distance matrix of our *observation × species* data frame, where species were columns, and rows were either surveys, or gardens, and species percent coverage values were centered, and Euclidean distances were applied. Sites with greater variance values indicate greater dispersion, and therefore greater beta diversity. Calculations were performed using the “betadisper” command in the “vegan” R package (Anderson et al. 2006; Oksanen et al. 2024). To calculate beta diversity of only wild or cultivated taxa, we generated new *observation × species* data frames with only those respective taxa in each.

To address Q1 and test the relationship between urbanization and beta diversity, we used linear regression models with beta diversity as the response and urbanization as the predictor at both spatio‐temporal scales (survey and garden), and was run for the full community, wild species, and cultivated species to address Q3. As beta diversity values ranged from 0 to 1 and exhibited a degree of heteroscedasticity, we used a beta error distribution for our models. To address Q2 and determine the similarities of community compositions between Berlin and Munich gardens, we paired a Non‐metric Multi‐Dimensional Scaling (NMDS) ordination with a vector analysis. The NMDS was run using the same distance matrix used in the beta diversity calculations (therefore Euclidean distances). Once the NMDS was created, we conducted a vector fitting analysis, which is a regression analysis that fits environmental variables onto the axes of the NMDS. This fitting method helps to determine if there are any statistically significant associations between NMDS axes and environmental variables via a permutation test (permutations = 999).

City identity (Berlin or Munich) and urban identity (more urban or more rural) were used as categorical predictors for our vector analysis. Here, urban identity was determined via a cut‐off of 50% imperviousness surrounding the garden, which split the dataset into 15 more urban gardens and 18 more rural gardens. Finally, to test the contribution of wild and cultivated taxa to overall beta diversity (Q4), we ran regression models with full community beta diversity as the response and either wild or cultivated beta diversity as the predictor. Contribution was determined via the *R* ^2^ of the models, with higher *R* ^2^ values indicating greater contribution to overall beta diversity. All statistical analyses were carried out using the statistical software R (version 4.1.2, R Core Team [2021]). Regressions used the R package “betagreg” (Cribari‐Beta and Zeileis 2010), the NMDS and vector analysis used the “vegan” package, and graphs and maps were produced using the “sf”, and “tidyverse” packages (Pebesma and Bivand 2023; Oksanen et al. 2024; Wickham et al. 2019).

## 3 Results

### 3.1 Beta Diversity and Urbanization Relationships

**FIGURE 2:**
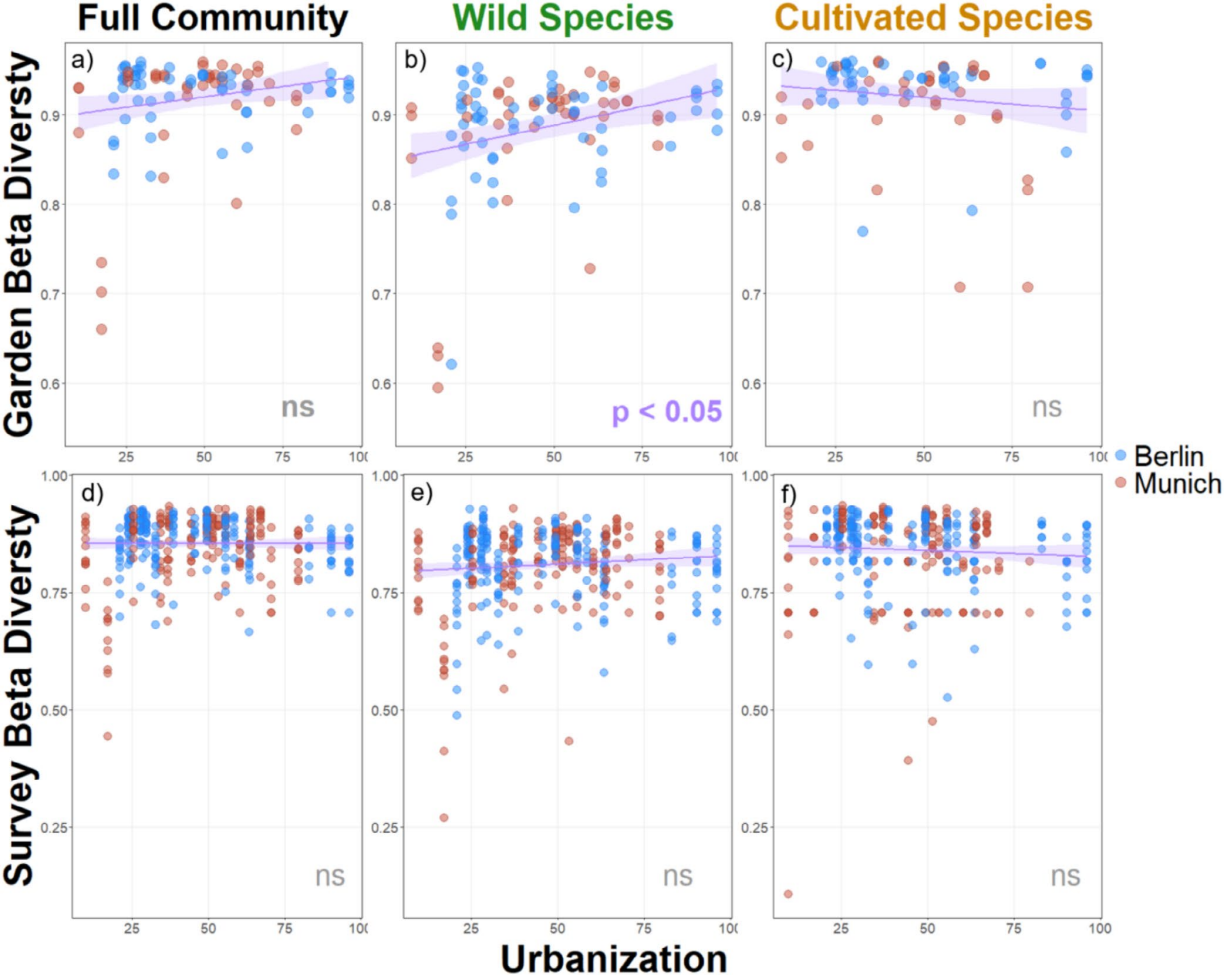
Relationship between urbanization and beta diversity. The top row shows (a through c) the relationship between urbanization and beta diversity at the garden level, and the bottom row (d through f) shows the relationship at the survey level. In the first column is using the entire plant community is used, while the second column shows only the wild species, and the final column shows the cultivated plant species. Urbanization and wild species beta diversity are significantly correlated at the garden level (*p* = 0.02, *R* ^2^ = 0.05), but all other relationships are non‐significant.

There were no significant relationships between urbanization and beta diversity within the full species community or the cultivated species communities at either the survey or garden level (Figure 2). Alternatively, beta diversity of wild communities increased along an urbanization gradient at the garden level (Figure 2, model outputs provided in Data S3). Here, we observed a roughly 10% increase in beta diversity of wild plant communities in urban gardens compared to rural gardens. At both spatio‐temporal scales and for all species types, the directionality of relationships was consistent across cities, indicating similar patterns of urbanization's influence on plant community beta diversity in Berlin and Munich.

### 3.2 Community Compositions

**FIGURE 3:**
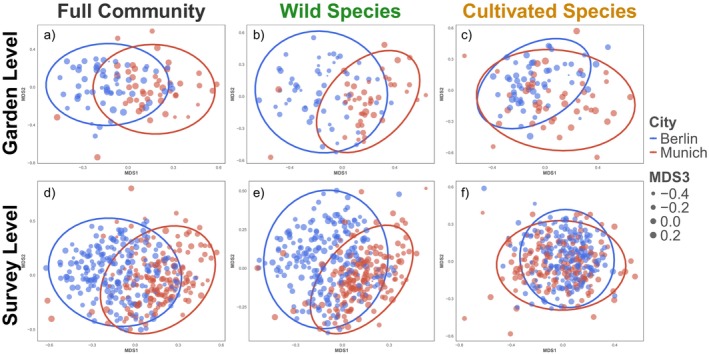
Plant community composition. The top row shows (a through c) plant communities at the garden level, while the bottom row (d through f) shows communities at the survey level. Berlin communities are shown in blue while Munich communities are shown in red. In all instances the community compositions were significantly different between the two cities (statistics provided in Data S4).

Community composition of the two cities, Berlin and Munich, was significantly different from each other at both the survey and garden levels (Figure 3). City identity explained 21% of plant community‐wide variation at the garden level, and 18% at the survey level. Urbanization did not significantly explain plant species community composition at the garden or survey level (Figure 3 and Data S3). Among wild plant species, community composition also differed by city at both levels, explaining 22% of the variation at the garden level, and 19% at the survey level (Figure 3). Urbanization did not influence wild plant species community composition at either spatio‐temporal scale (Data S3). For cultivated plant species, community composition was also significantly different between cities; however, the explained variation was much lower—city identity only explained 1% of the variation at the survey level, and 9% at the garden level. Additionally, urbanization significantly explained cultivated plant species composition; however, only explaining 4% of variation at both scales (Figure 3). Data S4 shows the ellipses of both cities' urban and rural gardens visualizing the lack of differentiation between urban and rural community compositions in either city.

### 3.3 Wild and Cultivated Taxa Contributions to Overall Beta Diversity

**FIGURE 4:**
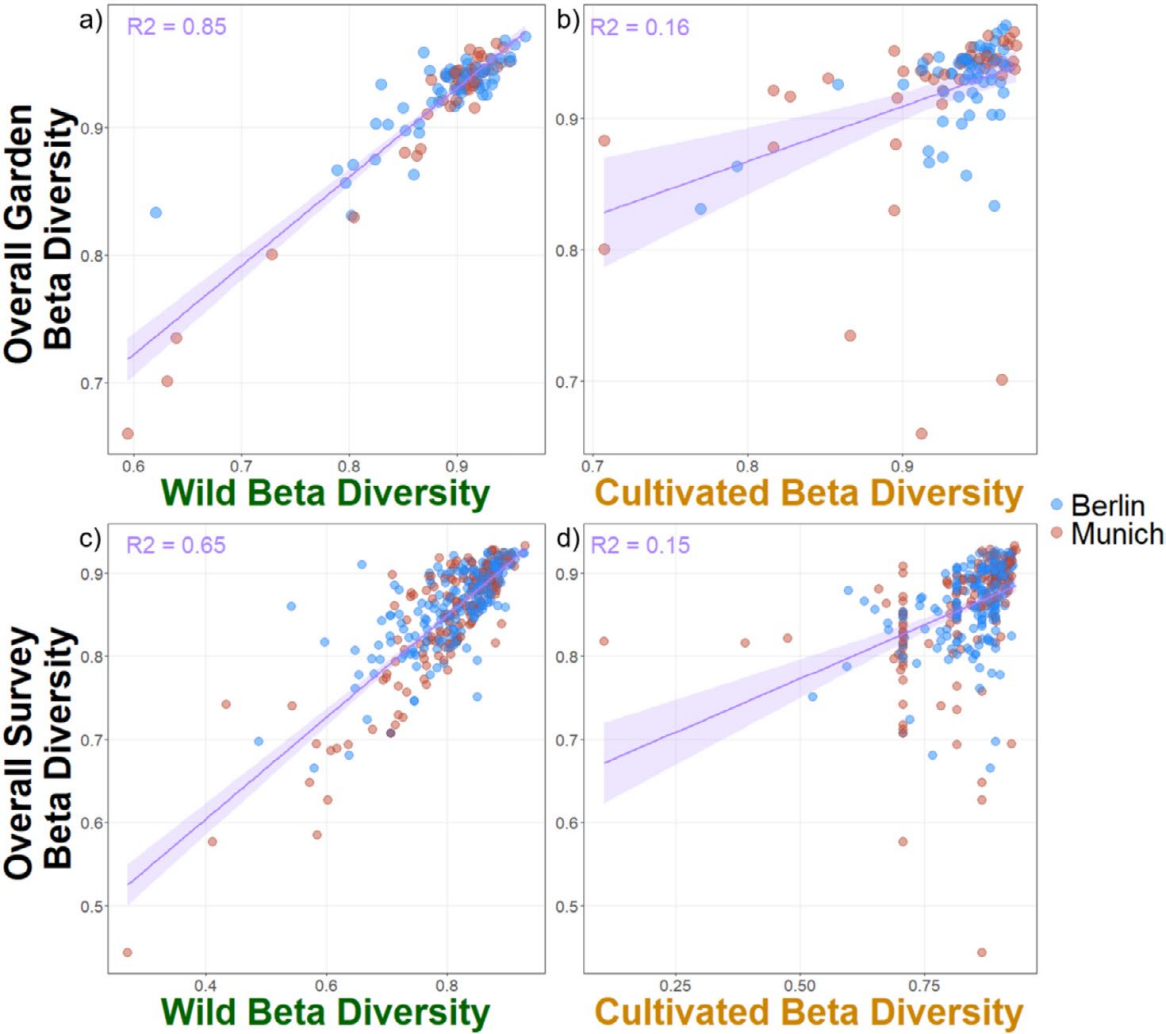
Contribution of wild and cultivated species to overall beta diversity. The top row (a and b) shows values at the garden level, and the bottom row (c and d) shows values at the survey level. Berlin communities are shown in blue while Munich communities are shown in red. While all relationships are significant (*p* < 0.01), *R* ^2^ values show that wild species are contributing more to overall beta diversity than cultivated species are.

At the garden level, wild species contributed highly to overall beta diversity, explaining 85% of the variation in overall beta diversity (Figure 4, *p* < 0.001, *R* ^2^ = 0.85, *t* = 24.34). Similarly, at the survey level, wild species contributed strongly to overall beta diversity, though slightly less than at the garden level (Figure 4, *p* < 0.001, *R* ^2^ = 0.66. *t* = 26.51). Cultivated species, however, contributed less to overall beta diversity, with lower *R* ^2^ and *t*‐values at both levels (Figure 4 and Data S3).

## 4 Discussion

Contrary to predictions posed by the Urban Biotic Homogenization (UBH) hypothesis, we found that plant communities in urban community gardens were distinct between two spatially independent cities, and they did not become more homogeneous with increasing urbanization. These findings refute the two central tenets of the UBH, namely that urban communities will be more like one another in comparison to their rural counterparts, and that beta diversity—an indicator of spatio‐temporal heterogeneity—will decrease with increasing urbanization (McKinney 2006). We found these patterns at two spatio‐temporal scales examined, and across both cities, indicating a consistent effect regardless of city. Wild plant species in these systems were largely driving these effects: wild plant species communities were increasingly diverse in more urban gardens, with beta diversity and urbanization being positively correlated, representing increased biotic differentiation. These wild, or spontaneous, plant species were also more important for explaining overall beta diversity in the system—much more so than cultivated plant species. In contrast, cultivated plant species communities were largely homogeneous across the two cities, providing support for UBH within this group. These results suggest that wild plants, if allowed to live next to their cultivated neighbors, can play a key role in maintaining and increasing biotic heterogeneity within and across urban ecosystems.

### 4.1 No Loss in Biotic Heterogeneity Along Urbanization Gradients

**FIGURE 5:**
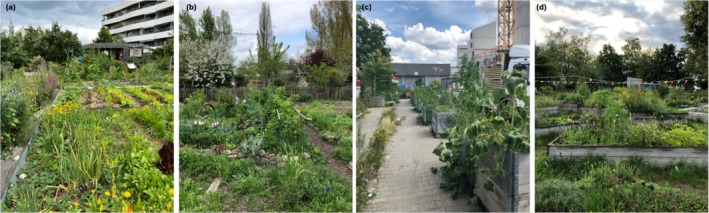
Gardens. All panels represent photos of the community gardens from our study system; panel a and b are of community gardens in Munich, while panels c and d show community gardens in Berlin.

The lack of a negative relationship between beta diversity, our metric of biotic heterogeneity, and increasing urbanization is likely due to a combination of natural and cultural processes. Community gardens are a rather novel urban ecosystem that represent an accumulation of historical land use legacies with contemporary active management through plant species cultivation by a diverse mix of people (Lin et al. 2018; Lin and Egerer 2020; Philpott et al. 2020). They are what Kowarik (2005) terms “Nature of the Third Kind”, as a construction of cultivated (horticultural) and wild plant species that can make them diverse and heterogeneous. These systems may have altered substrates, relics of earlier horticultural plantings, a proliferation of introduced cultivated species, as well as immigration and colonization of wild, native plant species with relatively longer dispersal distances (Kowarik 2005). In our system (and likely like other cities), community gardens often arise on vacant lands, next to remnant forests, along railroad easements, as well as on top of parking lots and concrete slabs (Figure 5) (Egerer et al. 2024). This variety in site histories and neighboring habitats can support meta‐community dynamics whereby community gardens support diverse, heterogeneous plant communities. A 2019 study of Berlin's wild plant communities found 213 endangered plant species within the city and found that “novel ecosystems” (i.e., vegetation patches not including parks, remnant vegetation, forests, etc.) harbored the highest number of endangered species populations (Planchuelo et al. 2019). These novel ecosystems are well dispersed throughout the urban landscape, and likely serve as steppingstone habitats, operating as population sinks and sources, facilitating meta‐community dynamics in the city and supporting wild plant populations. In older European cities, such as Berlin, these remnant vegetation patches and novel ecosystems often exist in the city center, while surrounding suburban areas tend to be dominated by more recent development that is quite different from the city center. This may explain our finding of increased wild plant beta diversity with increasing urbanization, as there is an abundance of these novel habitats to support wild plant species. Additionally, in the city center where space is highly limited, there is increased competition for space, and a frequently shifting environment that may alter microhabitats. For example, a large building was constructed next to one of our urban gardens in 2021, dramatically increasing the shade in the garden—something many gardeners have commented on.

In addition to meta‐community dynamics at the city level, beta diversity in these gardens is likely also a function of variation in gardener practices (Liere et al. 2020; Pearsall et al. 2017). The continuous cultivation of gardens, the accumulated effects of gardening practices, and the turnover of individual gardener members within the gardener community, may mean that the mix of cultivated and wild plants is highly dynamic through short‐term succession processes, regardless of degrees of urbanization. Thus, an individual community garden may significantly change from year‐to‐year due to a change in gardening activity and cultivation practices, such as the propensity to “weed” or remove spontaneous vegetation surrounding cultivated vegetation. This may explain why we observed larger increases in wild species beta diversity at the garden level than at the survey level, as garden level likely better encompasses these year‐to‐year changes. Such social processes are generally independent of urbanization context and create new and highly dynamic forms of urban nature (Liere et al. 2020).

### 4.2 Berlin and Munich Plant Communities Are Unique From One Another, Especially Among Wild Species

We found divergence in the community composition of the full plant communities between cities, especially so for the wild plant community. Furthermore, differentiation in the full plant community is largely influenced by wild plant species who drive city differentiation rather than city homogenization. Thus, we find no evidence for the UBH to explain patterns in the full plant communities nor wild plant communities in the gardens. However, we do see strong similarities in the cultivated plant communities across the two cities. The lack of support for the UBH hypothesis in wild plants, but support in cultivated plants suggests that wild plant communities are structured by the environmental factors inside these gardens, and the distinct natural and urban development histories of these two cities. Both climatic and urban planning histories can influence the composition of wild plant species that can survive in urban landscapes (de Barros Ruas et al. 2022; Sukopp and Wurzel 2000; Schmidt et al. 2014), and as Berlin and Munich harbor unique histories in both respects, it comes as no surprise that the two cities harbor unique wild plant communities. There is evidence of historical processes influencing modern plant communities elsewhere—a recent study of wild plant communities across nine major cities in China found their diversity to be influenced by both current state and historical processes of urbanization (Gao et al. 2023).

Berlin's urbanization history may make the city unique regarding habitat heterogeneity at the landscape scale, as well as the wild plant species pool that influence the potential for alpha and beta diversity within and across habitat patches (von Der Lippe et al. 2020). First, the Berlin metropolitan region is a biodiversity rich region of Germany, with several protected natural areas (European Natura 2000 sites) and a variety of habitat types within the city borders (von Der Lippe et al. 2020). This includes remnant natural habitats (woodlands, grasslands, meadows, ponds and wetlands), novel ecosystems and hybrid ecosystems (Kowarik 2005), all of which host unique plant communities including endangered “Red Listed” species mentioned above (Planchuelo et al. 2020). Thus, the accumulation of many different ecosystems means that the species pool of wild plants can be rather large. Such endangered wild species are even highly present within novel urban ecosystems like gardens (Planchuelo et al. 2019). Novel ecosystems such as urban gardens and restored ecosystems can increase the proportion of non‐native species introduced by people. Second, Berlin's regional urban planning practices are an incorporation of both Soviet‐style urban planning which incorporated the inclusion of fields, woodlands and agricultural lands and Western‐style urban planning (Moss 2020). As Berlin unified in the 1990s and further urbanized after the Cold War, these different urbanization patterns between the West and former East Berlin were incorporated into the expanding urban metropolitan area to result in diverse urban green spaces (von Der Lippe et al. 2020). There are still ‘Brachen’ (vacant) habitats and remnant habitat patches that contribute largely to the plant species pool of the city and are hotspots of Red Listed endangered species (Planchuelo et al. 2020). Munich, on the other hand, has been long developed, densified, and there are fewer fallow lands or wildlands within the metropolitan region (Gelfond et al. 2020). Although Munich is considered a very green city due to large green spaces such as the English Garden, its green spaces are concentrated and still relatively highly managed. Current urban expansion is toward surrounding agricultural lands, and there is less incorporation of wildlands. As such, Berlin's wild species pool is 50 species larger than Munich's (498 and 447 in each city respectively in our dataset). These unique forms of urban development influence the habitat patches of today, and therefore the urban plant communities.

### 4.3 Gardeners Likely Play a Large Role in Shaping Both the Wild and Cultivated Species' Beta Diversity in Urban Gardens

While urban planning histories may explain large differences in wild plant communities between Berlin and Munich, cultural gardening differences between the two cities may also explain differences. Berlin has a long and rich history of urban ecology and nature conservation centered around its “Brachen” or vacant ruderal habitats (Sukopp 2008; Stoetzer 2018), where people are proud of the city's greenness and wildness. This acknowledgement of the City's diversity is integrated into a contemporary Charta's to protect urban nature in the city (berlin.de/sen/uvk/natur‐und‐gruen/charta‐stadtgruen). Such top‐down policies are committed to protecting wild nature in the city, and thus to an extent the native wild plant species pool. This culture of wild, ruderal diversity may lead to reduced weeding of community gardens, and therefore greater flourishing of those populations both within community gardens, and across the city. In sum, the UBH hypothesis may be difficult to mainstream across cities of different natural histories and urbanization histories (Lokatis et al. 2023). These factors continue to shape the distribution of plant species, particularly those that are wild and spontaneous, within urban ecosystems.

Community gardens are the confluence of different management practices that stem from various individuals' motivations to garden, as well as established top‐down rules and regulations that guide management decisions within the community of gardeners (Lin and Egerer 2020). Gardeners have various motivations to participate in community gardens and to cultivate distinct plant species (Draper and Freedman 2010; Philpott et al. 2020; Sonti and Svendsen 2018), ranging from food production to connecting to nature, and to socializing and learning with fellow gardeners. Differences in motivations may drive differences in plant communities. Our previous work has shown that gardeners in our study system are motivated to implement pro‐biodiversity practices through their gardening activity, particularly due to positive emotions (Sturm et al. 2021). Gardeners also reported the desire to contribute to something meaningful such as nature conservation. These gardeners may either introduce native wild plants to their garden plots or allow wild spontaneous plants to grow within their plots or along edges, facilitating their survival within the gardens. We see this across urbanization gradients; even in highly urbanized gardens, we may find over a dozen species of wild plant species within cement block crevices (Figure 5c). These crevice plant communities provide hidden potential for the diversity of species that may thrive in community gardens, even those that are novel ecosystems established on concrete.

Cultivated plant communities lend further insight into the socio‐cultural drivers of plant communities as they are the direct result of gardeners' motivations, backgrounds, and management practices (Avolio et al. 2021; Kendal et al. 2012, 2023). If socio‐cultural differences between gardeners and cities were present, we would expect significant differences between city identity and cultivated plant communities. However, we found no clear evidence of differences in cultivated plant communities across cities or across urbanization gradients, providing evidence for the UBH when it comes to cultivated plant species. This may be because the species pool of cultivated plants is consistent in planting the same fruit and vegetable plants that yield high reward—tomatoes, squash, beans, and cole (Brassica) crops (Philpott et al. 2020; Lin et al. 2024). While there are few exceptions that may drive community differences, for example, the introduction of soybean, ginger, or chickpea by experimental and courageous gardeners (Seitz et al. 2022), the results indicate that regardless of gardener diversity or city context, distinctions in cultivated plant diversity are lost when accumulated across space and time. This indicates that biotic homogenization could occur across longer periods of time, especially among the cultivated plant communities, and should be monitored. This will become increasingly important as urban areas expand and natural areas are converted into spaces dominated by cultivated vegetation. As such, land managers and gardeners will play a larger role in biotic filtering and community composition; therefore a consideration of biotic homogenization among both cultivated and wild species should be made. Additionally, future research investigating the role that land managers play in cultivated plant community compositions is warranted in other urban habitat types such as urban parks, forests, and grasslands, as there are ample opportunities to combat urban biotic homogenization here.

### 4.4 Wild Species Facilitate Biotic Heterogeneity, and Should Therefore Be Supported in Urban Areas

Wild plant species influence the overall heterogeneity in the plant communities of these urban community gardens, and gardeners can promote urban biodiversity by maintaining and increasing wild plant species populations. Thus, from an applied perspective, encouraging gardeners to foster habitat for not only their cultivated plants but also native, wild, and spontaneous species allows the facilitation of rich and unique biodiversity in urban landscapes—especially among plant communities (Kendal et al. 2012, 2023). Because we show here that cultivated species may drive homogenization over space and time, incorporating more wild and native species is critical. However, this does require a certain trade‐off in land use and therefore a negotiation of people's motivations to garden, the shared use of available space, and acceptance of wildness in their gardens. These negotiations can focus on the benefits of wild plant species for urban agriculture—for example, recent studies have shown increases in plant richness are associated with strong increases in pollinator abundance and diversity, and in turn increases in fruit production in urban environments (Cohen et al. 2021; McDougall et al. 2022). Additionally, arguments can be made to focus on the human health benefits of increased biodiversity in urban landscapes, where the literature is rich (Dean et al. 2011; Hedblom et al. 2014; Ulmer et al. 2016). Through human facilitation, gardens may foster positive, synergistic relationships between cultivated and wild species, and in doing so increase habitat heterogeneity. By supporting both wild and cultivated species in gardens across urbanization gradients, gardeners may be able to combat instances of urban biotic homogenization. Particularly, as we show that wild species contribute the most to beta diversity, leaving space for wild species to establish, or even seeding in native, non‐cultivated plants has the potential to increase the beta diversity of urban ecosystems.

## 5 Conclusion

The high diversification of plant communities, and specifically of wild plant communities, within and across urban community gardens lends more support for urban heterogeneity than urban homogenization. In systems where moderate levels of disturbance are present, or where mixed land use practices occur, UBH may not be supported. It may be therefore valuable to re‐consider the UBH hypothesis, and where it is maintained, and where it is not. In other words, what are the required ecosystem features and social‐ecological mechanisms that prevent UBH and promote urban heterogeneity? How can we harness findings to inform ecosystem to landscape scale management to support urban biodiversity? There are many types of urban ecosystems, many of which are often overlooked in field studies, that can harbor unique, rare, and heterogeneous plant and animal communities. While the preservation of urban remnants is, of course, critical for species of conservation concern, such as red‐listed species, and maintaining urban ecosystem functions, community gardens can also play an important role in maintaining or supporting urban heterogeneity within and across urban landscapes. With proper management and planning tools, as well as educational resources for practitioners on the value of for example, native and wild plants, we can create more diverse and resilient urban ecosystems and landscapes for people and nature.

## Author Contributions

**Aaron N. Sexton:** conceptualization (equal), formal analysis (lead), project administration (supporting), visualization (lead), writing – original draft (lead), writing – review and editing (equal). **Monika Egerer:** conceptualization (equal), data curation (equal), funding acquisition (equal), methodology (equal), project administration (equal), resources (equal), supervision (equal), writing – original draft (supporting), writing – review and editing (equal). **Felix Conitz:** data curation (equal), methodology (equal), writing – review and editing (equal). **Ulrike Sturm:** conceptualization (supporting), data curation (equal), methodology (equal), writing – review and editing (equal).

## Conflicts of Interest

The authors declare no conflicts of interest.

## Supporting information


Data S1.

Data S2.

Data S3.

**Data S4.** NMDS plots visualizing plant community composition in urban and rural community gardens across Berlin & Munich. The top panel shows the full community, the bottom left shows the wild, or spontaneous species, and the bottom right shows cultivated species. Blue points represent Berlin gardens, red points represent Munich gardens, and in both cities darker colors represent urban gardens, while lighter colors represent rural gardens.

## Data Availability

All data and code used for the analyses in this manuscript have been made publicly available at the following archive: https://doi.org/10.5281/zenodo.15560000.
