# Supplementary figures and images for: Wild Plants Drive Biotic Differentiation Across Urban Gardens
Source: Ecol Evol. 2025 Jun 4;15(6):e71527. doi: 10.1002/ece3.71527 (PMC12137618; doi:10.1002/ece3.71527)

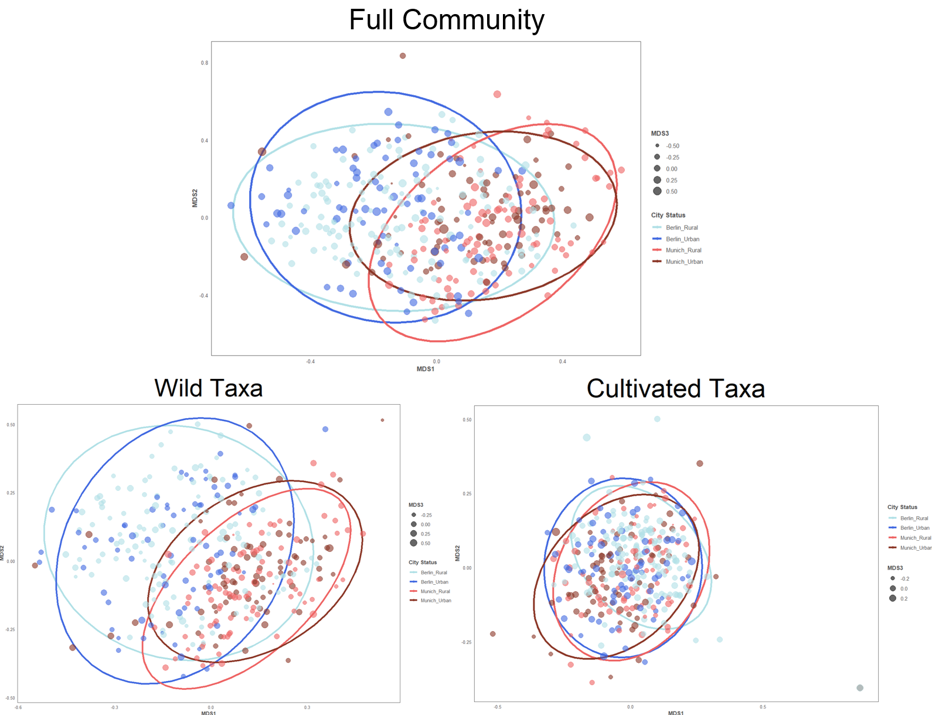

Supplement: Supplementary file 4 — Data S4. NMDS plots visualizing plant community composition in urban and rural community gardens across Berlin & Munich. The top panel shows the full community, the bottom left shows the wild, or spontaneous species, and the bottom right shows cultivated species. Blue points represent Berlin gardens, red points represent Munich gardens, and in both cities darker colors represent urban gardens, while lighter colors represent rural gardens. [file ECE3-15-e71527-s003.png]
